# Apathy in Parkinson's disease is related to executive function, gender and age but not to depression

**DOI:** 10.3389/fnagi.2014.00350

**Published:** 2015-01-15

**Authors:** Antonia Meyer, Ronan Zimmermann, Ute Gschwandtner, Florian Hatz, Habib Bousleiman, Nadine Schwarz, Peter Fuhr

**Affiliations:** ^1^Clinical Neurophysiology, Department of Neurology, Hospital of the University of BaselBasel, Switzerland; ^2^Epidemiology and Public Health, Swiss Tropical and Public Health Institute, University of BaselBasel, Switzerland

**Keywords:** apathy, Parkinson's disease, executive functions, depression, gender, age

## Abstract

Deficits in executive functions occur in up to 93% of patients with Parkinson's disease (PD). Apathy, a reduction of motivation and goal-directed behavior is an important part of the syndrome; affecting both the patients as well as their social environment. Executive functions can be subdivided into three different processes: initiation, shifting and inhibition. We examined the hypotheses, (1) that apathy in patients with Parkinson's disease is only related to initiation and not to shifting and inhibition, and (2) that depression and severity of motor signs correlate with apathy. Fifty-one non-demented patients (19 = female) with PD were evaluated for apathy, depression and executive functions. Executive function variables were summarized with an index variable according to the defined executive processes. Linear regression with stepwise elimination procedure was used to select significant predictors. The significant model (*R*^2^ = 0.41; *p* < 0.01) revealed influences of initiation (*b* = −0.79; *p* < 0.01), gender (*b* = −7.75; *p* < 0.01), age (*b* = −0.07; *p* < 0.05) and an age by gender interaction (*b* = 0.12; *p* < 0.01) on apathy in Parkinson's disease. Motor signs, depression and level of education did not influence the relation. These results support an association of apathy and deficits of executive function in PD. Initiation strongly correlates with apathy, whereas depression does not. We conclude, that initiation dysfunction in a patient with Parkinson's disease heralds apathy. Apathy and depression can be dissociated. Additionally, apathy is influenced by age and gender: older age correlates with apathy in men, whereas in women it seems to protect against it.

## Introduction

Apathy is defined as a primary loss of motivation, a loss of interest and reduced goal-directed behavior (Levy and Dubois, 2006; Starkstein et al., 2012). Apathy is a predictive factor for cognitive deterioration in Parkinson's disease (PD) (Dujardin et al., 2009); and has significant impact on quality of life (Zesiewicz et al., 2010; Chaudhuri et al., 2011).

Executive dysfunctions are highly prevalent in PD, affecting up to 93% of the patients, depending on the disease stage (Emre, 2003) and correlate with apathy (Pluck and Brown, 2002; Zgaljardic et al., 2007; Dujardin et al., 2009).

Executive functions comprise several processes which may be independently impaired (Miyake et al., 2000; Drechsler, 2007; Stuss and Alexander, 2007). Drechsler (2007) identified three executive processes: initiation, inhibition and shifting. Initiation is defined as the ability to start intentional actions self-motivated. Impairments in this process are associated with tests, in which patients are self-generating a processing speed or starting an action. Inhibition is characterized by the ability to suppress a reaction. Patients with impaired inhibition are showing impulsive response behavior and a high level of distractibility. Shifting means the ability to relocate the focus of attention from one to another target. It is reduced if a subject shows cognitive inflexibility and tests measuring this executive process are characterized by changing criteria within the task (Drechsler, 2007).

Major depression is present in up to a third of all patients with PD (Reijnders et al., 2010) and overlaps with apathy (Pluck and Brown, 2002; Kirsch-Darrow et al., 2006; Starkstein et al., 2009). However, there is evidence, that the two syndromes can be dissociated (Levy et al., 1998; Kirsch-Darrow et al., 2006).

Apathy due to frontal lesions shows a decreased performance in tests measuring the initiation process (Drechsler, 2007). We assume that dysfunction of initiation also predicts apathy in PD. Furthermore, we expected apathy to be related to depression. Several examinations have shown a relation between apathy and motor signs (Pedersen et al., 2010; Cubo et al., 2012), and therefore, we expected motor signs to be predictive for apathy. The aim of this study is to investigate the association of neuropsychological, psychiatric and motor factors with apathy in PD.

## Materials and methods

### Patients

Fifty-eight patients with PD were recruited between October 2011 and April 2013 from the movement disorders clinic of the Basel University Hospital or through advertisements. The patients were participants of a Cognitive training-study (Zimmermann et al., 2014) and underwent neuropsychological, psychiatric and neurological assessment. Inclusion criteria for the study were idiopathic PD according to UK Parkinson's disease Brain Bank Criteria (Gibb and Lees, 1988) and written informed consent. Patients were excluded if they had moderate or severe dementia (Mini Mental State Examination (MMSE) ≤ 24; Folstein et al., 1975), insufficient knowledge of the German language, other severe brain disorders, alcohol or drug dependency. For this study, the data of *n* = 51 patients (19 female, 32 male) has been analyzed. Seven patients were excluded because of having deep brain stimulation (*n* = 3), an unfulfilled apathy questionnaire (*n* = 3) or because German was not their native language (*n* = 1) (see Supplementary Figure 1). All patients were on dopaminergic medication and were tested in the ON state.

### Psychiatric and psychological assessment

#### Apathy

Apathy was measured with the German version of the Apathy Evaluation Scale (AES_D_; Lueken et al., 2006) filled out by a relative or a person close to the patients. This questionnaire consists of 18 items, which are evaluated on a four-point Likert scale, ranging from *not at all* (i.e., 0 points) to *a lot* (i.e., 3 points). For each patient, a total value was calculated with a maximum of 54 and minimal of zero point with higher values indicating more severe apathy.

#### Depression

Symptoms of depression were measured with a self-rating scale, the German version of Beck Depression Inventory II (BDI_D_; Hautzinger et al., 1995). The 21 items are answered on a four-point Likert scale, ranging from zero (e.g., *I do not feel sad*) to three (e.g., *I am so sad and unhappy that I can't stand it*). Based on the evaluation of each item, a total score was calculated. Maximum score is 63 and minimal score is zero, whereby higher values are indicating more severe depression.

### Neurological assessment

The severity of motor signs was assessed using Unified Parkinson's disease Rating Scale (UPDRS; Fahn and Elton, 1987), subscale III, applied by trained neurologists. This scale consists of 13 items which are evaluated on a four-point Likert scale. The patients are interrogated and examined about their motor issues (e.g., speech, mimicry, rest tremor, bradykinesia etc.). A total score is calculated with zero being minimal and 54 being the maximum value.

### Neuropsychological tests of executive functions and classification of variables

The classification of the variables follows Drechsler's (2007) definition of executive functions (see Table 1). The executive variables were classified each to one executive process by the consensus of two raters, and were then converted into z-scores and summarized to an index variable; initiation, shifting and inhibition. Additional information about the tests and analyzed variables is annexed in the Supplementary Material (see Supplementary, description of tests and measures).

**Table 1:** **Performance in tests measuring executive functions**.

**Executive process^a^**	**Test**	**Variable**	***Mdn [quantile]***
Initiation	Phonemic fluency	Correct answers	12 [10, 15]
	Semantic fluency	Correct answers	21 [17.5, 23]
	5 point test	Correct figures	23 [18.5, 28.5]
	TMT	TMT A	45 [37, 58.5]^b^
	Stroop	Naming colors	14 [13.7, 17]^b^
Shifting	mWCST	Perseverative errors	1 [0, 2.5]
	CVLT	Perseverative errors	2 [0.5, 3]
	TMT	TMT B/A (ratio)	2.9 [1.9, 2.7]
	Flexibility	Reaction time	4 [1, 9]^c^
Inhibition	Stroop	Interference	1.8 [1.6, 2.1]
	TMT	TMT B, errors	0 [0, 1]
	Divided attention	Errors	3 [1, 6]
	Working memory	Errors	5 [2, 7.5]

a *Classification according to Drechsler (2007). All medians (quantiles) represent raw values. Quantile refer to 25th and to the 75th percentile. TMT, Trail Making Test; mWCST, modified Wisconsin Card Sorting Test; CVLT, California Verbal Learning Test*.

b *Seconds*.

c *Milliseconds*.

#### Initiation

The initiation process was measured with:

- Semantic Fluency (Morris et al., 1989)
- Phonemic Fluency (Thurstone and Thurstone, 1948)
- 5 Point Test (Regard et al., 1982)
- Trail Making Test (Reitan, 1958)
- Stroop Test (Stroop, 1935)

#### Shifting

The shifting process was measured with:

- Modified Wisconsin Card Sorting Test (Nelson, 1976)
- California Verbal Learning Test (Delis et al., 1987)
- Trail Making Test (Reitan, 1958)
- Flexibility [Test of Attentional Performance (TAP); Zimmermann and Fimm, 2007]

#### Inhibition

The inhibition process was measured with:

- Stroop Test (Stroop, 1935)
- Trail Making Test (Reitan, 1958)
- Divided Attention (TAP: Zimmermann and Fimm, 2007)
- Working Memory (TAP: Zimmermann and Fimm, 2007)

### Statistical analysis

Level of statistical significance was set to *p* < 0.05. R version 3.0.1 was used for the analysis (R Core Team, 2012). All variables were averaged over the patients and z-scored; variables indicating good performance in smaller values were inversed.

The apathy total score was not normally distributed (Kolmogorov Smirnow Test (KST): *p* < 0.01) therefore, this variable was square root transformed, leading to a non-significant *p*-value (*p* = 0.34) of the KST. After z-score transformation, the executive function variables were averaged per each executive process: initiation, shifting and inhibition. To check the validity of the sub processes of executive functions, the intercorrelations between all the collected z-transformed cognitive variables and internal consistency (Cronbach's alpha) were calculated. As defined by George and Mallery (2003), acceptable internal consistency was set at Cronbach's α of greater or equal to 0.70. A linear regression model with Akaike Information Criterion (AIC) based stepwise backwards elimination procedure (Venables and Ripley, 2002) was applied to select the relevant predictors of apathy. The analyses were performed using initiation, inhibition, switching, age, gender, education and subscale III of the UPDRS as potential predictors for AES_D_ total score. Multiple *R*^2^ (coefficient of determination) and F-statistics were used for characterization of the overall model. The R package relaimpo (Grömping, 2006) was used to evaluate the relative importance of the regressors. Relaimpo splits the explained variance (*R*^2^) according to the importance of the predictors (Grömping, 2006).

## Results

### Patients

Low average total scores of apathy and depression were found in our sample of 51 PD patients. According to the proposed cut-off score of 19 points in the AES_D_(Leentjens et al., 2008), 8% of the patients met the criteria for clinical relevant apathy. In 8% of the included patients, a total score in the BDI_D_ of ≥14 was observed. Therefore, most of the patients had sub-syndromal depression as defined by Schrag et al. (2007). Table 2 shows the properties of the sample.

**Table 2:** **Sample description**.

	***Mdn [quantile]*^a^**
**Patients (*n* = **51**, 19 Female)**	
Age (years)	67 [32, 73]
Education (years)	15 [13, 16]
AES_D_	6 [2, 13]
BDI_D_	8 [4, 11.5]
MMSE	29 [28, 30]
UPDRS, subscale III	14.5 [7.6, 21]
Hoehn and Yahr stage	2 [0, 2]
LED (mg/day)	600 [300, 947]
Disease duration (years)	4 [2, 6.5]

a *All values represent median [quantiles]. Quantiles refer to 25th and to the 75th percentile. AES_D_, Apathy Evaluation Scale; BDI_D_, Beck Depression Inventory; UPDRS, Unified Parkinson's Disease Rating Scale; LED, L-Dopa- Equivalent Dose*.

### Internal consistency of initiation, inhibition, and shifting

The internal consistency for initiation was Cronbach's α = 0.78. This finding indicates acceptable internal consistency for this executive process. In contrast, it was inadequate for inhibition (Cronbach's α = 0.48) and shifting (Cronbach's α = 0.41).

The significant model (*R*^2^ = 0.41; *p* < 0.01) revealed influences of initiation (*b* = −0.79; *p* < 0.01), gender (*b* = −7.75; *p* < 0.01), age (*b* = −0.07; *p* < 0.05) and an age by gender interaction (*b* = 0.12; *p* < 0.01) on apathy in PD. Motor signs, depression and level of education did not influence the relation.

### Predictors for apathy

**Figure 1:**
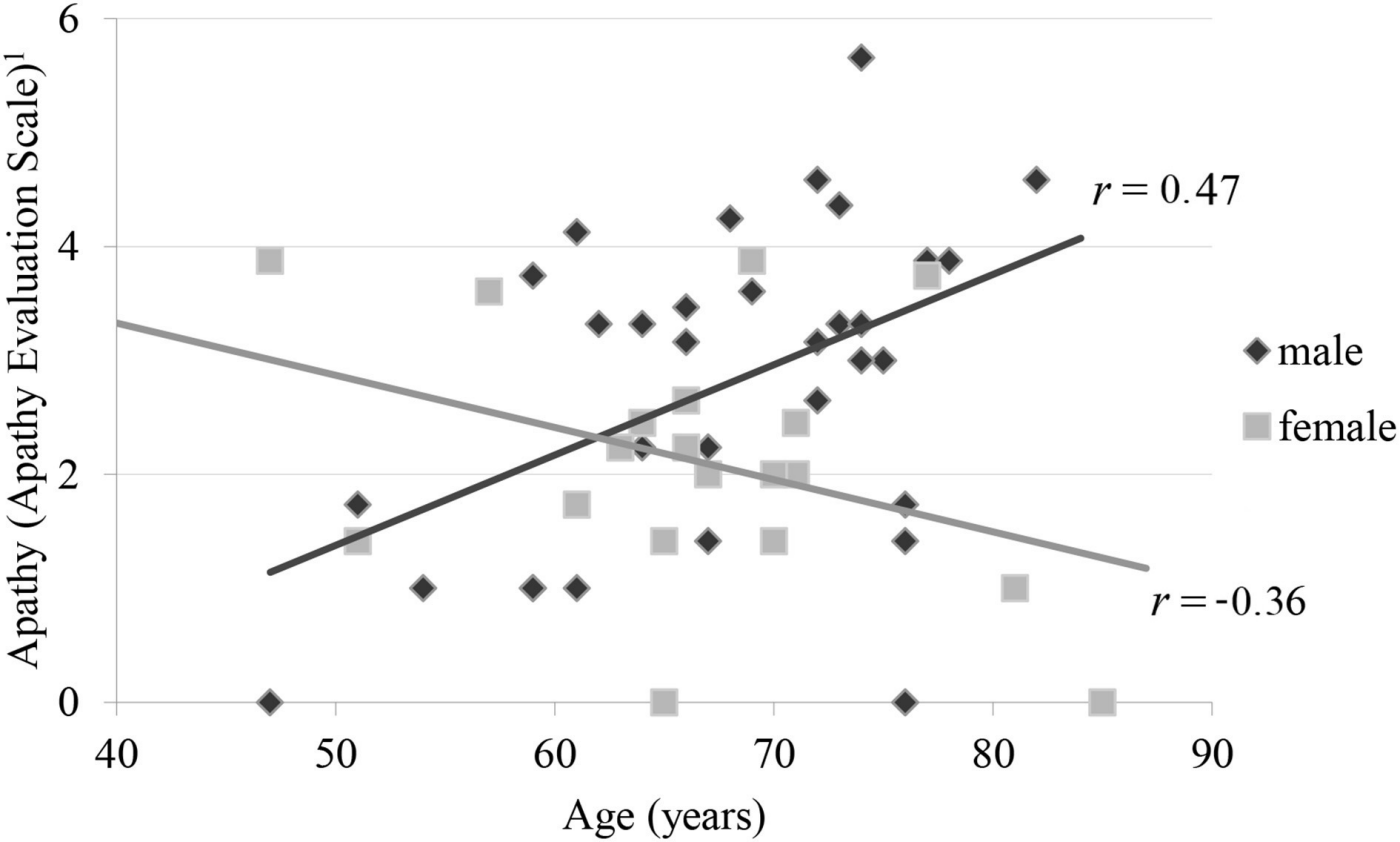
**Correlation of apathy and age by gender ^1^Apathy Evaluation Scale represents root transformed values**.

By stepwise elimination the following variables were excluded from the model: depression, inhibition, shifting and education. The parameters initiation (*b* = −0.79; *p* < 0.01), age (*b* = −0.07; *p* > 0.05), gender (*b* = −7.75; *p* < 0.01) and the interaction of age by gender (*b* = 0.12; *p* < 0.01) remained in the model explaining apathy. Furthermore, the UPDRS subscale III was not significant in the prediction of the AES_D_ total score (*b* = −0.01; *p* = 0.18), but remained in the model according to AIC. The overall model was significant (*p* < 0.01; *R*^2^ = 0.41); adjusted *R*^2^ = 0.35; *F*_(5, 44 = 6.24)_. According to Cohen (1992) an adjusted *R*^2^ of 0.35 can be interpreted as a strong effect. The calculation of the relative importance of the regressors in the model showed, that initiation and the interaction age by gender were the most important variables predicting apathy in PD. Initiation explained 17%, the age by gender interaction 15%, gender 6% and age 2% of the variance of apathy. The age by gender interaction showed that, in men, the AES_D_total score was positively correlated with age (*b* = 0.06; *p* < 0.05) while it was negatively correlated in women (*b* = −0.06; *p* < 0.05) (see Figure 1).

### Intercorrelations

**Figure 2:**
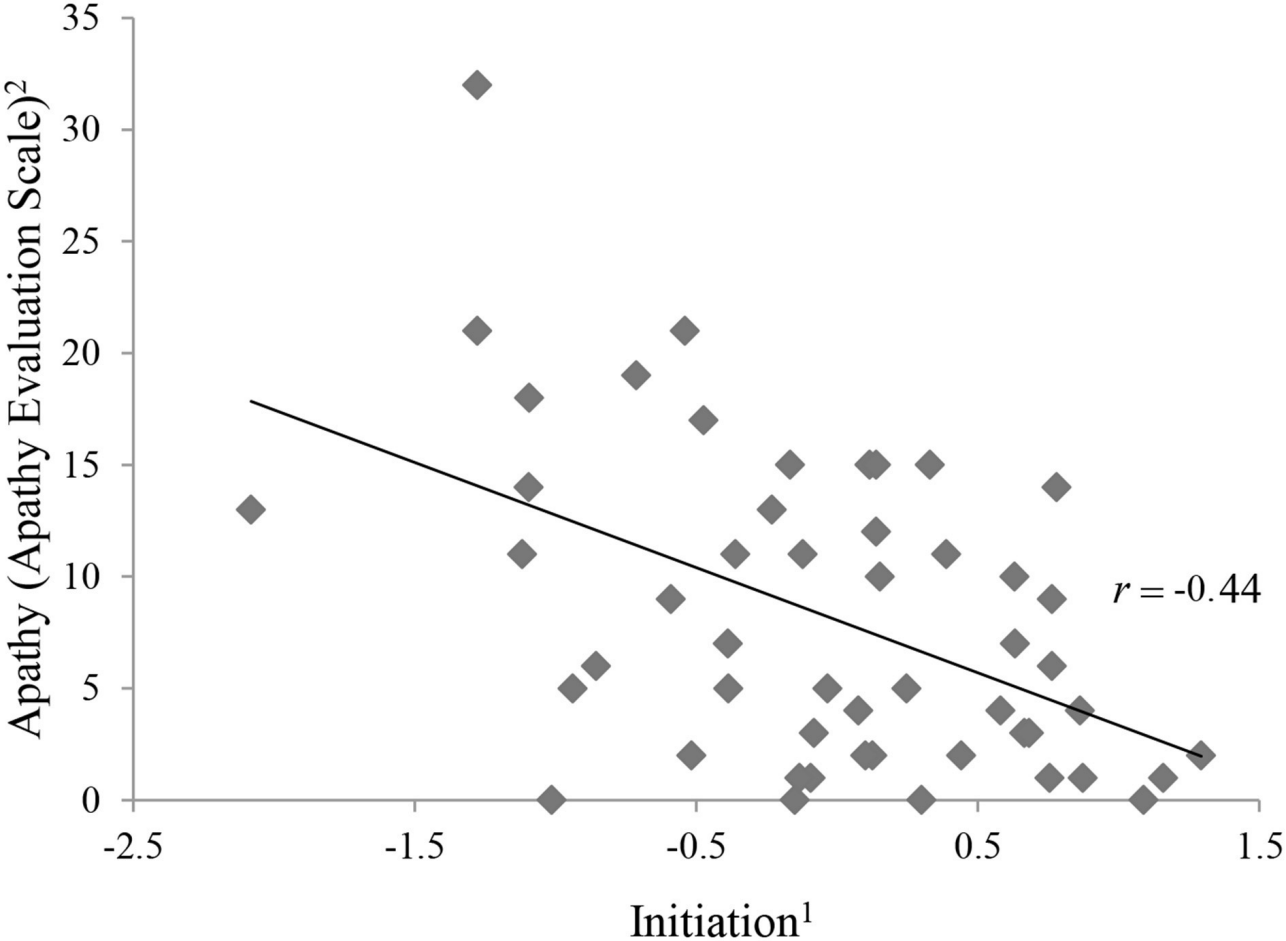
**Correlation between apathy and initiation.^1^The ability to start an intentional actions self-motivated. ^2^Apathy Evaluation Score represents raw values**.

Table 3 shows the intercorrelations of apathy and the executive functions variables. With exception of phonemic fluency, all variables categorized as initiation (Semantic Fluency, Five Point Test, Trail Making Test, Stroop Test) correlated significantly and negatively with apathy (see Figure 2).

**Table 3:** **AES_D_, Apathy Evaluation Scale; 5PT, Five Point Test; TMT, Trail Making Test; mWCST, modified Wisconsin Card Sorting Test; CVLT, California Verbal Learning Test; RT, reaction time**.

	**1**	**2**	**3**	**4**	**5**	**6**	**7**	**8**	**9**	**10**	**11**	**12**	**13**	**14**
1. AES_D_, total score	1													
**Initiation**	−0.44^**^													
2. S-words, correct words	−0.13	1												
3. Animals, correct words	−0.35^**^	−0.45^**^	1											
4. 5PT, correct figures	−0.36^**^	0.32^*^	0.56^**^	1										
TMT, A	−0.41^**^	0.18	0.43^**^	0.40^**^	1									
Stroopt test, naming colors	−0.35^**^	0.29^*^	0.42^**^	0.54^**^	0.58^**^	1								
**Shifting**	**−0.26**													
7. mWCST, perseverative errors	−0.25	−0.11	0.25	0.29^*^	0.26	0.09	1							
8. CVLT, perseverative errors	0.03	0.20	0.00	−0.02	−0.21	0.13	−0.22	1						
9. TMT, B/A (ratio)	−0.08	0.35^*^	0.37^**^	0.42^**^	0.04	0.24	0.25	0.00	1					
10. Flexibility RT	−0.24	−0.30^*^	0.37^**^	0.35^*^	0.31^*^	0.38^**^	0.12	−0.14	0.26	1				
**Inhibition**	**−0.23**													
11. Stroop test, interference	0.01	0.34^*^	0.23	0.24	−0.04	−0.16	−0.08	0.07	0.22	0.18	1			
12. TMT, part B errors	−0.25	0.35^*^	0.35^*^	0.22	0.36^*^	0.43^**^	0.22	−0.14	0.57^**^	0.35^*^	−0.03	1		
13. Divided attention, errors	−0.22	0.17	0.38^*^	0.27	0.26	0.33^*^	0.27	−0.02	0.44^**^	0.32^*^	0.14	0.38^**^	1	
14. Working memory, errors	−0.09	0.02	0.24	0.22	0.12	−0.07	0.31^*^	−0.08	0.38^**^	0.15	0.06	0.34^*^	0.23	1

** *p < 0.01*,

* *p < 0.050*.

## Discussion

Apathy is related to executive dysfunction in patients with PD but not to depression or motor signs. As hypothesized, the executive process of initiation is the most influential factor in this association. Incongruent with our initial hypothesis, neither depression nor motor symptoms significantly predict apathy. In our study, not all executive processes were predictive of apathy. Neither the single executive variables categorized as shifting, nor inhibition process variables nor the z-scored variables correlate with apathy (see Table 3).

In contrast to previous investigations (Aarsland et al., 2009; Pedersen et al., 2010; Varanese et al., 2011), the patients in this study were only slightly affected by apathy and depression. While this fact may be a limitation for the exclusion of weak but still existing relations, it helps to differentiate between distinct psychopathological syndromes; less severely affected patients are more likely to suffer from only one, instead of several disorders simultaneously.

The result regarding the relation between apathy and executive functions are in accordance with the existing literature (Pluck and Brown, 2002; Zgaljardic et al., 2007; Dujardin et al., 2009). Levy and Dubois (2006) propose dysfunctions in the prefrontal cortico-cortex-basal ganglia circuits to be involved in apathy. Dysfunctions in these circuits are also responsible for executive dysfunctions (Alvarez and Emory, 2006). Thus, it is possible that both initiation and apathy are caused by dysfunction in the prefrontal cortico-basal-ganglionic loop. However, some severely affected patients with PD do not suffer from apathy. From this, we can infer that dysfunction of the different cortico-basal loops (like the motor loop as opposed to the cingular-basal and other fronto-basal loop) is disparate.

An alternative explanation for the association between apathy and initiation might be that behavioral symptoms (i.e., lack of motivation, a loss of interest and reduced goal-directed behavior as defined by Levy and Dubois, 2006) are leading to a worse performance in initiation tasks.

Although “executive functions” are a widely accepted neuropsychological construct, the categorization of tests and variables measuring this dysfunction occur mostly in an arbitrary manner. In this study, we used the approach by Drechsler (2007) (i.e., initiation, inhibition, shifting) whereas Stuss and Alexander (2007) for example propose three different processes, i.e., *energization* (initiating and maintaining a response), *task setting* (ability of if-then setting and adjustment of contention scheduling) and *monitoring* (prolonged fore period effect and an increase of all subtypes of errors, including false negatives). This concept of executive processes is based on the analyses of the effects of lesions of the frontal lobes (Stuss and Alexander, 2007). Interestingly, the Drechsler's concept of initiation and Stuss' concept of energization seem to be congruent.

Contrary to our hypothesis we did not find an association between apathy and depression. This is consistent with the assumption that apathy and depression are distinct disorders (Kirsch-Darrow et al., 2006). It is also interesting to note that severity of motor signs is not related to apathy.

In this study, we find an unexpected interaction of age and gender on apathy in PD. The literature regarding the relation of gender and apathy in PD is incongruent. Some studies reported male patients to be more severely affected by apathy (Ready et al., 2004; Pedersen et al., 2010). In contrast the female patients in a multicenter review by Martinez-Martin et al. (2012) were predominantly affected by apathy compared to male patients. Kirsch-Darrow et al. (2006) have detected a significant association between age and apathy in PD, but they did not determine an effect of gender. None of the cited studies reported an interaction of age and gender on apathy in PD. It could possibly be related to the fact that apathy was rated by a relative or a person close to the patients. Thus, this age by gender interaction has not been reported so far and needs validation.

A limitation of this study is the insufficient internal consistency for shifting and inhibition. It might be criticized, that only initiation is predictive for apathy because it has highest internal consistency. However, this possibility is unlikely as the intercorrelations of the single executive variables composing shifting and inhibition were not significantly correlated with apathy, while the single variables used for initiation were. A further limitation of this study is the relatively small sample size. In addition, only a low number of patients are strongly pronouncing apathy respectively depression. Therefore, these results have to be replicated in a larger sample of patients with PD.

In conclusion, our data support an influence of initiation, gender and age on apathy in PD.

### Conflict of interest statement

The authors declare that the research was conducted in the absence of any commercial or financial relationships that could be construed as a potential conflict of interest.
