# Supplementary material for: Apathy in Parkinson's disease is related to executive function, gender and age but not to depression
Source: Front Aging Neurosci. 2015 Jan 15;6:350. doi: 10.3389/fnagi.2014.00350 (PMC4295432; doi:10.3389/fnagi.2014.00350)

Supplementary Material

Apathy in Parkinson`s disease is related to executive function, gender and age but not to depression

Antonia Meyer[1], Ronan Zimmermann[1], Ute Gschwandtner[1], Florian Hatz[1], Habib Bousleiman[1,2], Nadine Schwarz[1], Peter Fuhr[1*]

[1]Department of Neurology, Hospital of the University of Basel, Switzerland
[2] Epidemiology and Public Health, Swiss Tropical and Public Health Institute, University of Basel, Basel, Switzerland

**** Correspondence:***
Peter Fuhr, Department of
Neurology, Hospital of the
University of Basel, Petersgraben 4
4031 Basel, Switzerland
Peter.Fuhr@usb.ch

1. Supplementary descriptions of tests and measures

1.1. Initiation

The initiation process was measured with:

- Semantic and Phonemic Fluency (Morris *et al*., 1989; Thurstone and Thurstone, 1948), number of correct words
  Two versions of verbal fluency were completed, each during 60 seconds. In the semantic (Morris *et al*., 1989) task, the patients were asked to produce words of a specific category (i.e. *animals*). In the phonemic task (Thurstone and Thurstone, 1948) the patients had to produce words with a specific letter (i.e. *words with s*). In each test, the number of correctly generated words was collected.
- 5 Point Test (Regard *et al*., 1982), number of correct figures
  The 5 point test is a non-verbal fluency task. The patients received a sheet with 5x7 fields, each containing five points. They were instructed to generate different patterns by connecting at least two points during three minutes. The total number of correctly produced patterns was noted.
- Trail Making Test (TMT; Reitan, 1958), TMT A
  The TMT is a paper-pencil-test and includes two parts. In part A, the patients were instructed to connect the numbers *1 – 25* in ascending order as fast as possible without lifting the pen from the paper. The time to complete the test was recorded.
- Stroop Test (Stroop, 1935), naming colors
  The Stroop test includes three parts. In part one, naming colors, the patients were instructed to appoint as fast as possible the different colors of dots on a sheet. The time to complete this task was recorded.

1.2. Shifting

The shifting process was measured with:

- Modified Wisconsin Card Sorting Test (mWCST; Nelson, 1976), perseverative errors
  In the mWCST, four stimulus cards were presented to the patient, who has then to assign a stack of cards according to self-defined or investigator-defined target criteria (i.e. *number, color* or *form*). After each six correctly assigned cards, the participants were instructed to change the target criteria. For the shifting process, the number of perseverative errors was analyzed, expressing the wrongly maintaining of target criteria.
- California Verbal Learning Test (Delis *et al*., 1987), perseverative errors
  In the California Verbal Learning test, the patients were instructed to remember and reproduce sixteen items over several repeated trials and not to repeat several times a word in one trial (e.g. *pineapple, jeans, milk* etc.). Number of perseverative errors expresses the number of repetition made by the participant (i.e. *pineapple*, jeans, milk, *pineapple*).
- Trail Making Test (Reitan, 1958), part B/A (ratio)
  In part B of the TMT, the patient was instructed to connect alternately numbers and letter. Numbers, as in part A in ascending order, but also letters according the alphabet (i.e. *1 – A – 2 – B – 3 – C* etc.), the time to complete this task was recorded. The TMT B/A variable is expressing a time-measurement from part B relative to part A.
- Flexibility (Zimmermann and Fimm, 2007), reaction-time

Flexibility was measured with a computerized task (TAP: Zimmermann & Fimm, 2007). The patients had to switch their focus between simultaneously presented stimuli (e.g. *b and 4*) on a screen. The time reacting on the correct stimulus was analyzed for the shifting process.

1.3. Inhibition

The inhibition process was measured with:

- Stroop Test (Stroop, 1935), interference

  In the third part of the Stroop test, participants were asked to assign the ink of a written color-word and not to read the word (e.g. *red in BLUE ink*). The interference-variable is a ratio, between part one (e.g. *naming colors*) and part three.
- Trail Making Test (Reitan, 1958), number of errors

  This variable express the number of errors made by the patient in part B of the TMT.
- Divided attention (Zimmermann and Fimm, 2007), number of errors

  In this test, the patients were instructed to react simultaneously and as fast as possible on a visual (e.g. *scanning for a specific pattern on the panel*) and an auditory (e.g. *two following tones*) stimulus. The number of false positive errors means a reaction of the patient in absent of the described stimulus-pattern.
- Working memory (Zimmermann and Fimm, 2007), number of errors

  In this computerized working memory task, the patients saw a sequence of numbers on a panel and were asked to react, when the present number was identical to the second-last presented number. The analyzed variable expresses the reaction of the subject in absent of two following numbers, as defined.

2. Supplementary Figures

Figure 1: Enrollment and analysis of study patients.

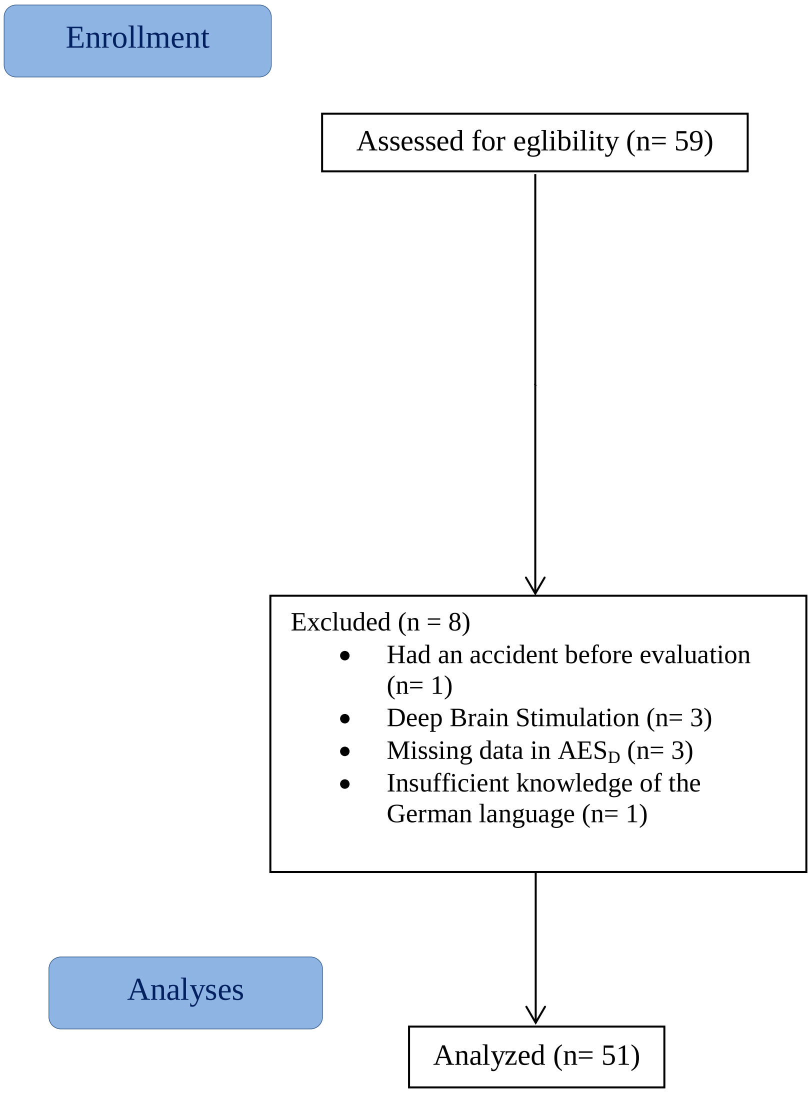

Supplement: Supplementary file 1 [file Presentation1.PDF]
